# Study of Podoplanin Expression in Head and Neck Squamous Cell Carcinoma

**DOI:** 10.30699/IJP.2022.547004.2809

**Published:** 2021-08-12

**Authors:** Anjali Mary Jibi, Vijaya Basavaraj

**Affiliations:** *Department of Pathology, JSS Medical College, JSS Academy of Higher Education and Research, Mysuru, Karnataka, India*

**Keywords:** Head and neck squamous cell carcinoma, Podoplanin, Lymph node metastasis

## Abstract

**Background & Objective:**

Head and Neck Squamous cell carcinoma (HNSCC) is one of the leading cancers worldwide. Cervical lymph node metastasis is the most adverse prognostic factor for patients with HNSCC. As there are no reliable factors in predicting lymph node metastasis, recent researchers focus on identifying various metastasis markers that will aid treatment selection. Podoplanin is a recent marker strongly associated with lymph node metastasis, aggressive tumor behavior, and poor prognosis. The expression of podoplanin in human squamous cell cancers and its association with cancer cell motility suggest a possibility that it could be used as a biomarker to predict lymph node metastasis. To study the expression of podoplanin in head and neck squamous cell carcinoma, determine its association with clinicopathological variables, and predict its use as a biomarker in predicting lymph node metastasis.

**Methods:**

The present study was conducted in a tertiary care hospital. Podoplanin expression was studied in 45 cases of HNSCC and its association with clinicopathological variables. The predictive power of podoplanin was further analyzed using univariate and multivariate logistic regression analysis. The positive and negative predictive values of podoplanin were determined concerning the presence or absence of lymph node metastasis.

**Results:**

Podoplanin expression is significantly associated with histological grade (*P*=0.03) and lymph node metastasis (*P*=0.01). In logistic regression analysis, podoplanin expression (Odds Ratio: 5.66, Confidence Interval: 1.23 -25.87, *P*=0.02) was a significant independent predictor of lymph node metastasis.

**Conclusion:**

**Our study demonstrates that podoplanin provides prognostic information and predicts lymph node metastasis which was consistent with our studies in the literature. Thus, podoplanin may help better stratify patients selected for elective neck node dissection in early tumor stages and clinically negative regional disease.**

## Introduction

Head and neck squamous cell carcinoma (HNSCC) is one of the leading cancers worldwide, increasing morbidity and mortality. Head and neck cancers rank sixth worldwide, with an annual incidence of more than 550,000 new cases (1). Most head and neck squamous cell carcinomas arise in the epithelial lining of the oral cavity, oropharynx, larynx, and hypopharynx (2). In India, oral squamous cell carcinoma (OSCC) accounts for increased mortality in men, reaching up to 22.9% of cancer-related deaths. Incidence is influenced by lifestyle, habits, demographics, and genetic factors. Despite recent advances in treatment, the survival rates of patients have not improved significantly (3).

Tumor metastasis to regional lymph nodes is a major prognostic indicator for disease progression (4). The presence of lymph node spread is important for the survival of patients with HNSCC because malignant cells preferentially metastasize to roughly 400 lymph nodes in the cervical region (5).There are no reliable factors available in predicting cervical lymph node metastasis. Hence, recent research focuses on identifying various markers for tumor progression that will facilitate treatment selection (1). Although several molecules and clinical studies have documented the relevance of hematogenous dissemination, little is known regarding the mechanisms through which tumor cells invade the lymphatic system. Investigations at the molecular level started around 10 years ago, and podoplanin was one of the first markers to be discovered in lymphatic endothelial cells. Thus, over the past few decades, podoplanin has been used to understand tumor behavior and progression in various carcinomas (6).

Podoplanin is a 38 k Da mucoprotein specifically expressed in the lymphatic endothelium. It was first discovered in puromycin-induced nephrosis on the surface of mouse podocytes. In normal human tissue, podoplanin is expressed in kidney podocytes, skeletal muscle, placenta, lung and heart, myofibroblasts of the breast and salivary glands, osteoblasts, and mesothelial cells, and in the basal layer of the human epidermis (7). In a knock-out animal model, it has been shown that podoplanin deficiency causes lymphovascular malformations, suggesting that podoplanin may have a role in lymphangiogenesis (8). With the advent of recent studies, podoplanin has been found in several carcinomas like squamous cell carcinoma, mesothelioma, and germ cell tumors (2). Transfection studies with cultured normal and cancer cells in vitro support that podoplanin expression in human cancers promotes migration and invasion of cancer cells and metastasis to regional lymph nodes in vivo (7).

D2-40 is one of the most commonly used monoclonal antibodies to demonstrate lymphovascular invasion and lymphatic differentiation in many tumors. They react to podoplanin by targeting the oncofetal M2A antigen, resulting in the membranous expression of tumor cells. This antibody's utility in podoplanin-expressing cancers has been demonstrated in previous literature (6).

The role of podoplanin in tumor invasion and metastasis raises the possibility that it could be used as a predictive marker for lymph node metastasis in HNSCC. Hence, the present study is undertaken to study the expression of podoplanin in HNSCC, determine its association with clinicopathological variables, and evaluate its use as a biomarker in predicting lymph node metastasis.

## Material and Methods

The study was conducted in a tertiary care hospital in South India. The study included 45 cases, both prospective and retrospective cases, for four years. The inclusion criteria were all radical specimens of head and neck cancers. Cases with additional treatment before surgery and adenosquamous cell carcinomas were excluded from the study. In retrospective cases all the clinical information was retrieved, and the sections were taken from the paraffin blocks stored in the department. In prospective cases, the clinical history was born, the tissue biopsies were kept for fixation, and the sections were prepared after routine processing. Subsequently, these sections were subjected to hematoxylin and eosin staining. Histological grading of the tumor was done according to Broder’s classification. Pathological TNM staging was done.

Tissue processing and immunohistochemical analysis were performed on three to four µm thick sections. The sections were placed on Poly – L – Lysine coated slides, air-dried, and antigen retrieval was done by Tris buffer in the pressure cooker. Peroxide block was applied for 10 minutes and washed with wash buffer for one minute. The sections were incubated with primary antibody D2-40 for 20 minutes. The bound antibody was visualized using a DAB - chromogen substrate. A section from the tonsil was taken as a positive control, whereas a section treated with Tris-buffer solution instead of the primary antibody was used as the negative control.

Podoplanin expression was scored as described by Hyun-Ii Kim* et al.*, and the positive tumor cells were quantified from zero to five as follows: - 0 for negative, 1 for 1- 10%, 2 for 11-30%, 3 for 31 to 50%, 4 for 51 to 80% and 5 for 81 to 100 %.The staining intensity was scored from zero to three: - 0= negative, 1= weak, 2= moderate and 3 = strong. German Immunoreactive Score (IRS) was obtained by multiplying quantity score and staining intensity scores. Scores above six were designated high podoplanin reactivity, and scores six and below were designated weak podoplanin reactivity**.**

## Results

Out of 45 patients, 28 (62%) were males (M), and 17 (38%) were females (F). The M: F ratio was 1.65:1 with a clear male preponderance. The patients were in the range of 36 to 80 years, with a mean age of presentation at 61(±12Standard deviation) years. The predominant number of patients was 51 to 60 years old, accounting for 31.1% of total cases. Twenty-nine cases (64.4%) gave a history of smoking or tobacco use, and the remaining sixteen (35.5%) cases were non-tobacco users. In the present study, 45% had a tumor in the larynx, followed by 42% in the oral cavity, and the remaining 13% were found in the hypopharynx. According to grade, 89% of tumors were less differentiated, and 11% were well-differentiated tumors. Among 45 cases, 23 had lymphovascular invasion (LVI), and six had perineural invasion (PNI).Out of 19 oral cancers, 21% of cases had a depth of invasion (DOI) less than five mm, 53% had a DOI ranging from five to ten, and 26% had a DOI of ten or greater than ten.The majority of the cases presented at Tumor (T) stages two and three (73% of total cases).Twenty-three (49%) cases had metastases to cervical lymph nodes at the time of presentation, while the remaining 22 cases (51%) had no metastasis. Of 23 metastatic lymph nodes, 11cases (48%) showed extra-nodal extension (ENE).

**Fig. 1:**
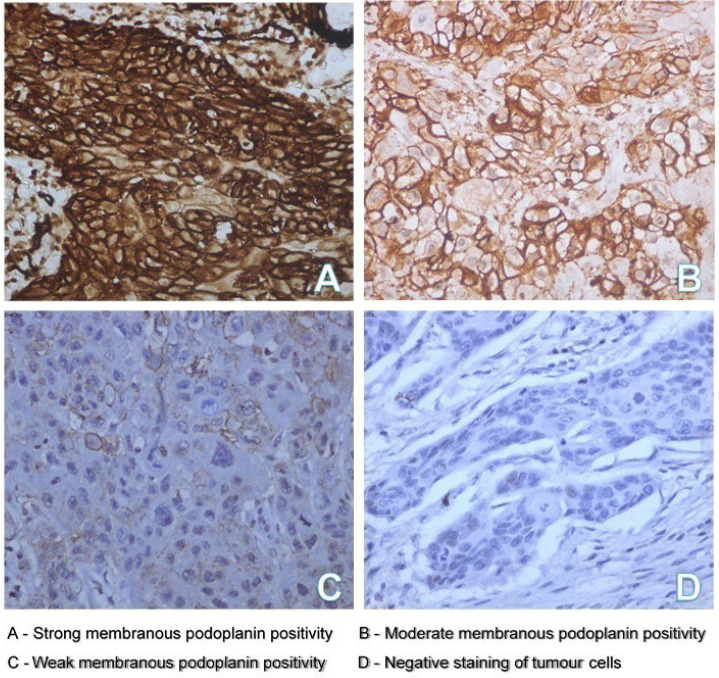
Staining intensity of tumor cells

Podoplanin expression of tumor cells was quantified based on the estimated percentage of positively stainedtumor cells. Eighteen cases (40%) showed podoplanin positivity in 31-50% of tumor cells, eleven cases (24%) showed positivity in51-80% of tumor cells, seven cases (16%) showed positivity in 1 – 10% of tumor cells, five cases (11%) showed positivity in 11-30% of tumor cells, and two cases (4%) showed positivity in >80% of tumor cells. The remaining two cases (4%) were negative for podoplanin. Immunohistochemical staining images showing the percentage of tumor cells involved are shown in Figure 1.

**Fig. 2:**
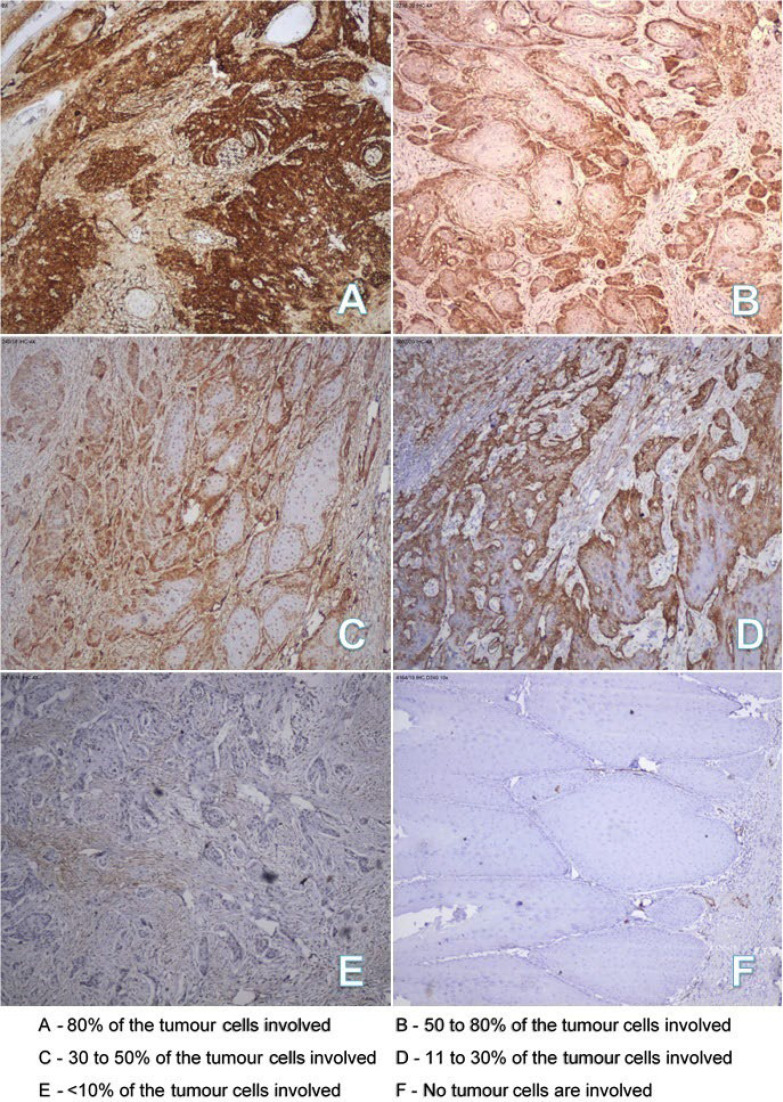
Staining intensity of tumor cells

The staining intensity of the tumor was scored as negative, weak, moderate, and strong. Nineteen cases (42.2%) showed strong staining intensity of the tumor cells, sixteen cases (36%) showed moderate staining intensity, eight cases (18%) showed weak staining intensity, and two cases (4.4%) showed negative staining of tumor cells. Immunohistochemical staining images showing the staining intensity of tumor cells are shown in Figure 2.

The IRS total score ranged from zero to fifteen. For statistical purposes, we divided the cases into two groups; those with seven or higher scores were designated high podoplanin reactivity, and zero to six scores were designated weak reactivity. Twenty-four cases (53%) showed high podoplanin reactivity, and twenty-one cases (47%) showed low reactivity.

The association between podoplanin expressions with clinicopathological variables of the 45 HNSCC patients is shown in Table 1. There was a significant association between high podoplanin expression and poor histological grade (*P*=0.03) and the presence of lymph node metastasis (*P*=0.01). There was a trend towards increased T stage of the tumor with high podoplanin expression, although not statistically significant. Depth of invasion (DOI) was measured for nineteen cases of oral cancers, and it was observed that podoplanin reactivity was higher in tumors with DOI greater than or equal to five millimeters than in tumors with a depth of invasion less than five millimeters. But we did not obtain a significant correlation (*P*=0.09).

Univariate and multivariate logistic regression analysis was used to find the predictors of lymph node metastasis. Univariate analysis of lymph node metastasis with clinicopathological variables and podoplanin expression are shown in Table 2. In univariate analysis, lymphovascular invasion (LVI) (*P*=0.01) and podoplanin expression (*P*=0.01) were significant predictors of lymph node metastasis. Significant variables in univariate analysis were used in multivariate logistic regression analysis, and it was observed that podoplanin (*P*=0.02) and LVI (*P*=0.01) were significant independent predictors of lymph node metastasis. Hence, podoplanin was associated with a fivefold increase in lymph node metastasis. Multivariate analysis of lymph node metastasis with significant variables from the univariate analysis is shown in Table 3.

In our analysis, podoplanin had a positive predictive (PPV) value of 67% and a negative predictive value (NPV) of 71%.

**Table 1:** Association between podoplanin expression and the clinicopathological variables in HNSCC

Variables		Number of Patients	Podoplanin Expression		P-value
			Low	High	
Age	<50	8	4 (19%)	4 (16.7%)	0.49
	51-70	27	14 (66.7%)	13 (54.2%)	
	>70	10	3 (14.3%)	7 (29.2%)	
Gender	Men	29	13 (61.9%)	16 (66.7%)	0.76
	Woman	16	8 (38.1%)	8 (33.3%)	
Tobacco	User	29	11(52.4%)	18 (75.0%)	0.13
	Non- user	16	10 (47.6%)	6 (25.0%)	
Tumor Site	Larynx	20	10 (47.6%)	10 (41.7%)	0.77
	Oral Cavity	19	9 (42.9%)	10 (41.7%)	
	Hypopharynx	6	2 (9.5%)	4 (16.7%)	
Histological Grade	Well	5	5 (23.8%)	0 (0.0%)	0.03*
	Moderate	36	14 (66.7%)	22 (91.7%)	
	Poor	4	2 (9.5%)	2 (8.3%)	
Lymphovascular Invasion	Absent	22	11(52.4%)	11(45.8%)	0.76
	Present	23	10 (47.6%)	13 (54.2%)	
Perineural Invasion	Absent	39	18 (85.7%)	21(87.5%)	1
	Present	6	3 (14.3%)	3 (12.5%)	
Tumor Stage	T1+T2	26	12 (57.2%)	14 (58.3%)	0.14
	T3+T2	19	9 (42.8%)	10 (41.6%)	
Nodal Metastasis	Absent	23	15 (71.4%)	8 (33.3%)	0.01*
	Present	22	6 (28.6%)	16 (66.7%)	
Extranodal Extension	Absent	32	16 (76.2%)	16 (66.7%)	0.53
	Present	13	5 (23.8%)	8 (33.3%)	

*P-value< 0.05 between the two categories for a given variable.

**Table 2:** Univariate analysis of lymph node metastasis with clinicopathological variables and podoplanin expression

Variables	Odds Ratio	P-value	95% Confidence Interval
Age (Years)	1.012962	0.61	0.9641462 - 1.06425
Sex	1.071429	0.91	0.3160005 – 3.632777
Duration of Illness	0.9924311	0.79	0.9387053 – 1.049232
Tobacco	3.116667	0.08	0.0580919 – 11.32001
Tumor Size (cms)†	0.8307692	0.76	0.2516279 – 2.74285
Grade‡	0.372549	0.41	0.35322 – 3.929363
LVI	6.095237	0.01^*^	1.674526 – 22.18652
Tumor Stage			
T1	1		
T 2	2.6	0.46	0.2054475 – 32.90378
T3	2	0.61	0.140766 – 28.41596
T4	0.8000001	0.88	0.0437073 – 14.64286
Podoplanin	4.999999	0.01^*^	1.402108 – 17.8303

* P-value < 0.05 between the two categories for a given variable. † Tumor size categories <2, 2 – 4and >=4 cms. ‡ Histological grade categories: well, moderately and poorly differentiated. LVI - Lymphovascular invasion**.**

**Table 3:** Multivariate analysis of lymph node metastasis with tobacco, LVI and podoplanin

Variables	Odds Ratio	P-value	95% Confidence Interval
Tobacco	2.261117	0.28	0.5039248 – 10.14566
LVI	7.597515	0.01^*^	1.687786 – 34.19998
Podoplanin	5.661772	0.02^*^	1.23892 – 25.87387

* P-value <0.05 between the two categories for a given variable. LVI-lymphovascular invasion

## Discussion

Nodal metastasis is an independent adverse prognostic factor in HNSCC. Previous analysis has shown that the overall survival of the patients decreases by 50% in metastasis to cervical lymph nodes (4). Podoplanin is a recent marker showing significant association with nodal metastasis and poor clinical outcome (8). In the previous studies, authors have reported that podoplanin expression was found at the invasive front of the tumor in more than 80% of human squamous cell carcinomas (9). A potential role of podoplanin in cancer progression was suggested due to its expression in various tumor model experiments, both in vitro and in vivo. Cueni Ln *et al.* found that expression of podoplanin on cancer cells increased lymph node metastasis in a human breast carcinoma xenograft model as well as in OSCC (10). They also revealed that podoplanin promotes metastasis to cervical lymph nodes by affecting several steps of tumor igenesis without altering primary tumor growth (10). Studies from multiple cancer biopsies, cultured human breast cancers and mouse model of carcinogenesis indicates that increased expression of podoplanin renders tumor cells motile and invasive, through actin cytoskeleton remodelling (11). Wound healing assays have also demonstrated the invasive property of podoplanin through interaction of VEGF-C in squamous cell carcinomas (7). Increased expression of podoplanin in invasive front of tumor and its consistent role in tumor progression suggest that podoplanin might have a role as a biomarker in predicting lymph node metastasis. 

Most of the tumors studied, showed podoplanin expression restricted to the peripheral layer of tumor nests. A diffuse pattern of expression was also observed. The peripheral layers of tumor showed increased expression of podoplanin because they have a higher proliferative capacity and the central tumor cells lack podoplanin expression as they are terminally differentiated cells. (1) Predominant staining patterns of tumor cells were membranous and less differentiated tumors also showed cytoplasmic staining. A total of 86% of cases showed increased podoplanin expression in the invasive front of the tumor. This observation was consistent with other studies reported in the literature (12,13). An IRS score was obtained for podoplanin expression as described by Hyun-Ii Kim* et al. *Twenty four cases (53%) showed high podoplanin reactivity and twenty one cases (47%) showed low podoplanin reactivity. Studies show no uniformity regarding the range of IRS score. Due to the inconsistency in methods of scoring podoplanin; incongruity exists between studies reporting podoplanin as a prognostic marker of HNSCC (13,14,15).

Age and differences in gender did not significantly correlate with podoplanin expression which is well documented in the literature (13,15). Predominant number of patients was in the age group between 51 to 60 years. The slight male preponderance may be due to higher susceptibility for smoking in males. According to the hospital based cancer registers in India, Head and neck cancers accounts for 29.8% to 50.4% of all cancers in males and 11-22% of all cancers in females (16). Tobacco was an important factor for tumor initiation and prognosis of HNSCC. Hashibe M *et al. *mentioned in their study that tobacco was an important factor for tumor initiation and prognosis of HNSCC (17). In the present study, there was a higher prevalence (64.4%) of tobacco use as observed by other studies (18,19). We also observed that, significant number of tobacco users (75%) had high podoplanin expression when compared to non-tobacco users. But the difference did not reach statistical significance. A similar observation was also observed in other studies (17).

Depending on the geographic area of occurrence, a difference has been noted in the most common site of occurrence of SCC in head and neck region (20). Oral and oropharyngeal SCC is common in India, Southeast Asia, China and Taiwan (20). In the present study, most common site was larynx and oral cavity, followed by hypopharynx. There was no significant statistical correlation with podoplanin expression and tumor site. 

Podoplanin expression increased with the grade of the tumor. There was a significant statistical association between tumor grade and podoplanin reactivity which was consistent with other studies (15,19). In the present study, well-differentiated carcinoma showed no expression and less differentiated carcinomas showed diffuse expression of podoplanin. It has been proposed that the expression of podoplanin focally in the peripheral layer of the tumor nest may indicate reduced biological aggressiveness than the diffuse pattern of podoplanin expression (12).

Experimental observations have proved that podoplanin expression increases cancer cell motility, thereby enhancing local invasion, as reflected by the advanced pathological T stage of human SCC with high levels of podoplanin expression (21). In our study there was a trend, although not significant, towards increased T stage with high podoplanin expression and a similar finding was also demonstrated in other studies (22,23).

An important feature of the malignant behavior of tumor cells is its capability to metastasize to various organs (24). Lymph node metastasis is associated with poor survival in patients with HNSCC (8). It was suggested that podoplanin can promote invasion via two pathways. Wicki *et al* in his study on human squamous cell carcinoma and in animal model of insulinoma, showed that podoplanin is involved which is independent of epithelial mesenchymal transition (EMT) pathway.However, there is also evidence that podoplanin can promote single cell invasion in human oral squamous cell carcinoma through EMT pathway(11).Through EMT pathway, podoplanin induces phosphorylation of ERM (Ezrin, Radixin, Moesin) proteins to activate RhoA stimulating actin polymerization leading to formation of filopodium, thereby enhancing cell migration and invasion(11). Through non-EMT pathway, podoplanin mediates direct activation of ERM by downregulating RhoA which leads to decreased formation of stress fibres (25). Platelet aggregation is known to influence tumor metastasis by protecting of tumor cells during their transit through the bloodstream. Podoplanin induces platelet aggregation and further accelerate the process of metastasis via induction of matrix metalloproteinases (6). Fifteen cases (66.7%) with nodal metastasis showed high podoplanin expression and 16 cases (71.4%) with no nodal metastasis showed low podoplanin expression. Thus, metastasis to lymph nodes was statistically significant when associated with podoplanin and our findings were consistent with other studies in literature (8,23). However, a few studies did not find any significant correlation between podoplanin expression and nodal metastasis (19,24).

Extranodal extension also has a major impact on the prognosis of head and neck cancers (26). Out of 23 cases with lymph node metastasis, 13 cases (59%) had ENE. Podoplanin expression was higher in those with ENE, but the difference did not reach statistical significance. Few studies have also demonstrated a similar finding (2,27). Relationship of podoplanin with ENE needs to be investigated further in larger sample size.

Depth of invasion is a significant predictor of subclinical nodal metastasis in early oral squamous cell carcinomas (28). Muhammed F *et al* in their study showed that depth of invasion greater than 10 mm had a risk of local recurrence of 40% and nodal metastasis of 53% (29).In oral cancers, we observed that podoplanin expression increased with depth of invasion, although not significant. Previous studies have emphasised on the role of podoplanin in tumor invasion, but no other studies demonstrated a correlation between podoplanin expression and depth of invasion (7).

The presence of LVI and PNI in oral cancers has a significant impact on survival outcome of patients (30). Studies have emphasised that podoplanin has a potential role for tumor progression and lymphovascular invasion (9). PNI and podoplanin are independent predictors of lymph node metastasis and poor survival (31). The association of podoplanin with LVI and PNI was not statistically significant as observed in other studies (27,28).

We also studied the expression of podoplanin with inflammatory cells in tumor microenvironment based on an observation made by Kunita* et al.* They observed that expression of podoplanin was induced by inflammatory cytokines secreted by the proinflammatory cells in the tumor microenvironment. When pro-inflammatory cells interpreted cytokine-mediated signal transduction in SCC cells, podoplanin expression reduces and with it tumor invasion (32). Hamada *et al* demonstrated that inflammation is associated with podoplanin expression. However, in our study, there was no significant association between podoplanin expression and inflammatory response. Further studies are required to emphasize the importance of inflammatory response on tumor growth (15).

LVI and podoplanin expression were significant predictors of lymph node metastasis in our univariable analysis. There was a trend although not significant for increased risk of lymph node metastasis among tobacco users. Previous have demonstrated a role of tobacco in tumor progression and poor clinical outcome (33). In our multivariable analysis, LVI and podoplanin expression were independent significant predictors of lymph node metastasis. Podoplanin was associated with a fivefold rise in lymph node metastasis. In our analysis, podoplanin had a PPV of 67% and NPV of 71%. Our data was consistent with other studies in the literature (15,34). Huber *et al.*, studied podoplanin expression in 120 patients with squamous cell carcinoma of the oral cavity or oropharynx and demonstrated that podoplanin is a significant predictor of lymph node metastasis (Odds Ratio: 2.70, 95% Confidence Interval: 1.11–6.54, *P*=0.028) (34). Hamada *et al.*, studied 99 patients with squamous cell carcinoma of oral cavity and demonstrated that podoplanin was a significant predictor of lymph node metastasis (Odds Ratio: 3.47, 95% Confidence Interval: 1.18‑10.2, *P*=0.005). They obtained PPV of 44% and NPV of 88% for podoplanin expression (15). LVI is another significant predictor of lymph node metastasis and tumor recurrence in cancers (35). However, its presence might be underrated in small biopsies due to reduced tumor sampling. There are no other definitive microscopic features or tumor markers to predict nodal metastasis on such small biopsy materials. Hence, podoplanin is one such marker which can be used to predict nodal metastasis in small biopsies (36). So, patients with clinically negative nodes but has increased podoplanin expression in the tumor cells, should be considered for elective neck dissection to prevent the risk of nodal metastasis. 

## Conclusion

Predicting lymph node metastasis by clinical or pathological parameters is difficult in patients presenting in early stages, based on which elective neck dissection or radiotherapy is undertaken (28). Also, there are no definitive pathological features or immunohistochemistry to predict the presence of neck nodal metastasis or to provide prognostic information on small biopsy materials (36). Accurate prediction of the prognosis of the newly diagnosed patient can assist the physician in patient counseling, clinical decision-making, and quality management. Our study demonstrates that high podoplanin expression predicts lymph node metastasis and aggressive tumor behavior. Thus, podoplanin can help stratify patients for elective neck dissection selection in early tumor stages and clinically negative nodal disease. 

There is increasing evidence that podoplanin might contribute to tumor growth, invasion, and hematogenous spread. Experimental studies suggest that podoplanin may be targeted both on its intracellular and extracellular domains to alter the motility of tumor cells. Thus, there is a potential role of monoclonal anti-podoplanin antibody in targeted therapy to prevent metastasis in the coming future, thereby improving the clinical outcome in patients with HNSCC.

The limitation of this study was the small sample size. Hence, we recommend that podoplanin expression in HNSCC be conducted in large samples at multiple centers for a better understanding of the utility of podoplanin as a prognostic biomarker.

## Conflict of Interest

The authors declared no conflict of interest.

## Funding

None.

## References

[1] Prasad B, Kashyap B, Babu GS, Kumar GR, Manyam R (2015). Expression of podoplanin in different grades of oral squamous cell carcinoma. Ann med health sci res.

[2] Kreppel M, Scheer M, Drebber U, Ritter L, Zöller JE (2010). Impact of podoplanin expression in oral squamous cell carcinoma: clinical and histopathologic correlations. Virchows Arch.

[3] Logeswari J, Malathi N, Thamizhchelvan H, Sangeetha N, Nirmala SV (2014). Expression of podoplanin in oral premalignant and malignant lesions and its potential as a biomarker. Indian J Dent Res.

[4] Wei WI (2002). Commentary: Head and neck carcinomas in the developing. BMJ-BRIT MED J.

[5] Zhang Z, Helman JI, Li LJ (2010). Lymphangiogenesis, lymphatic endothelial cells and lymphatic metastasis in head and neck cancer-a review of mechanisms. Int J Oral Sci.

[6] Raica M, Cimpean AM, Ribatti D (2008). The role of podoplanin in tumor progression and metastasis. Anticancer Res.

[7] Wicki A, Christofori G (2007). The potential role of podoplanin in tumour invasion. Br J Cancer.

[8] Kim HY, Rha KS, Shim G, Kim JH, Kim JM, Huang SM, Koo BS (2015). Podoplanin is involved in the prognosis of head and neck squamous cell carcinoma through interaction with VEGF-C. Oncol Rep.

[9] Cirligeriu L, Cimpean AM, Raica M, Doroş CI (2014). Dual role of podoplanin in oral cancer development. in vivo.

[10] Cueni LN, Hegyi I, Shin JW, Albinger-Hegyi A, Gruber S, Kunstfeld R, Moch H, Detmar M (2010). Tumor lymphangiogenesis and metastasis to lymph nodes induced by cancer cell expression of podoplanin. Am J Pathol.

[11] Wicki A, Lehembre F, Wick N, Hantusch B, Kerjaschki D, Christofori G (2006). Tumor invasion in the absence of epithelial-mesenchymal transition: podoplanin-mediated remodeling of the actin cytoskeleton. Cancer cell.

[12] Logeswari J, Malathi N, Thamizhchelvan H, Sangeetha N, Nirmala SV (2014). Expression of podoplanin in oral premalignant and malignant lesions and its potential as a biomarker. Indian J Dent Res.

[13] de Vicente JC, Santamarta TR, Rodrigo JP, García-Pedrero JM, Allonca E, Blanco-Lorenzo V (2015). Expression of podoplanin in the invasion front of oral squamous cell carcinoma is not prognostic for survival. Virchows Arch.

[14] Sgaramella N, Lindell Jonsson E, Boldrup L, Califano L, Coates PJ, Tartaro G, Lo Muzio L, Fåhraeus R, Colella G, Dell'AversanaOrabona G, Loljung L (2016). High expression of podoplanin in squamous cell carcinoma of the tongue occurs predominantly in patients≤ 40 years but does not correlate with tumour spread. J PatholClin Res.

[15] Hamada M, Ebihara Y, Nagata K, Yano M, Kogashiwa Y, Nakahira M, Sugasawa M, Nagatsuka H, Yasuda M (2020). Podoplanin is an efficient predictor of neck lymph node metastasis in tongue squamous cell carcinoma with low tumor budding grade. Oncol Lett.

[16] Dhull AK, Atri R, Dhankhar R, Chauhan AK, Kaushal V (2018). Major risk factors in head and neck cancer: a retrospective analysis of 12-year experiences. World J Oncol.

[17] Hashibe M, Brennan P, Benhamou S, Castellsague X, Chen C, Curado MP, Maso LD, Daudt AW, Fabianova E, Wünsch-Filho V, Franceschi S (2007). Alcohol drinking in never users of tobacco, cigarette smoking in never drinkers, and the risk of head and neck cancer: pooled analysis in the International Head and Neck Cancer Epidemiology Consortium. J Natl Cancer Inst.

[18] Kawaguchi H, El-Naggar AK, Papadimitrakopoulou V, Ren H, Fan YH, Feng L, Lee JJ, Kim E, Hong WK, Lippman SM, Mao L (2008). Podoplanin: a novel marker for oral cancer risk in patients with oral premalignancy. J ClinOncol.

[19] Kanliada D, Coskunpinar E, Orhan KS, Oltulu YM, Celik M, Eren A, Yaylim I, Deger K (2014). Investigation of biomarker in laryngeal carcinomas. J Clin Lab Anal.

[20] Znaor A, Brennan P, Gajalakshmi V, Mathew A, Shanta V, Varghese C, Boffetta P (2003). Independent and combined effects of tobacco smoking, chewing and alcohol drinking on the risk of oral, pharyngeal and esophageal cancers in Indian men. Int J Cancer.

[21] Inoue H, Miyazaki Y, Kikuchi K, Yoshida N, Ide F, Ohmori Y, Tomomura A, Sakashita H, Kusama K (2012). Podoplanin expression during dysplasia-carcinoma sequence in the oral cavity. Tumor Biol.

[22] Huber GF, Fritzsche FR, Züllig L, Storz M, Graf N, Haerle SK, Jochum W, Stoeckli SJ, Moch H (2011). Podoplanin expression correlates with sentinel lymph node metastasis in early squamous cell carcinomas of the oral cavity and oropharynx. Int J Cancer.

[23] Yuan P, Temam S, El‐Naggar A, Zhou X, Liu DD, Lee JJ, Mao L (2006). Overexpression of podoplanin in oral cancer and its association with poor clinical outcome. Cancer.

[24] Almeida AD, Oliveira DT, Pereira MC, Faustino SE, Nonogaki S, Carvalho AL, Kowalski LP (2013). Podoplanin and VEGF-C immunoexpression in oral squamous cell carcinomas: prognostic significance. Anticancer Res.

[25] Osazuwa-Peters N, Boakye EA, Chen BY, Tobo BB, Varvares MA (2018). Association between head and neck squamous cell carcinoma survival, smoking at diagnosis, and marital status. JAMA Otolaryngol Head Neck Surg.

[26] Cueni LN, Hegyi I, Shin JW, Albinger-Hegyi A, Gruber S, Kunstfeld R, Moch H, Detmar M (2010). Tumor lymphangiogenesis and metastasis to lymph nodes induced by cancer cell expression of podoplanin. Am J Pathol.

[27] Mermod M, Bongiovanni M, Petrova TV, Dubikovskaya EA, Simon C, Tolstonog G, Monnier Y (2017). Correlation between podoplanin expression and extracapsular spread in squamous cell carcinoma of the oral cavity using subjective immunoreactivity scores and semiquantitative image analysis. Head Neck.

[28] Kane SV, Gupta M, Kakade AC, D'Cruz A (2006). Depth of invasion is the most significant histological predictor of subclinical cervical lymph node metastasis in early squamous carcinomas of the oral cavity. Eur J SurgOncol (EJSO).

[29] Faisal M, Abu Bakar M, Sarwar A, Adeel M, Batool F, Malik KI, Jamshed A, Hussain R (2018). Depth of invasion (DOI) as a predictor of cervical nodal metastasis and local recurrence in early stage squamous cell carcinoma of oral tongue (ESSCOT). PLoS One.

[30] Jardim JF, Francisco AL, Gondak R, Damascena A, Kowalski LP (2015). Prognostic impact of perineural invasion and lymphovascular invasion in advanced stage oral squamous cell carcinoma. Int J Oral Maxillofac Surg.

[31] Cañueto J, Cardeñoso‐Álvarez E, Cosano‐Quero A, Santos‐Briz Á, Fernández‐López E, Pérez‐Losada J et al (2017). The expression of podoplanin is associated with poor outcome in cutaneous squamous cell carcinoma. J CutanPathol.

[32] Kunita A, Baeriswyl V, Meda C, Cabuy E, Takeshita K, Giraudo E, Wicki A, Fukayama M, Christofori G (2018). Inflammatory cytokines induce podoplanin expression at the tumor invasive front. Am J Pathol.

[33] Jethwa AR, Khariwala SS (2017). Tobacco-related carcinogenesis in head and neck cancer. Cancer Metastasis Rev.

[34] Huber GF, Fritzsche FR, Züllig L, Storz M, Graf N, Haerle SK, Jochum W, Stoeckli SJ, Moch H (2011). Podoplanin expression correlates with sentinel lymph node metastasis in early squamous cell carcinomas of the oral cavity and oropharynx. Int J Cancer.

[35] Kozłowski M, Naumnik W, Nikliński J, Milewski R, Łapuć G, Laudański J (2011). Lymphatic vessel invasion detected by the endothelial lymphatic marker D2-40 (podoplanin) is predictive of regional lymph node status and an independent prognostic factor in patients with resected esophageal cancer. Folia HistochemCytobiol.

[36] Dumoff KL, Chu CS, Harris EE, Holtz D, Xu X, Zhang PJ, Acs G (2006). Low podoplanin expression in pretreatment biopsy material predicts poor prognosis in advanced-stage squamous cell carcinoma of the uterine cervix treated by primary radiation. Mod Pathol.

